# Deficiency in KPNA4, but Not in KPNA3, Causes Attention Deficit/Hyperactivity Disorder like Symptoms in Mice

**DOI:** 10.3390/genes16060690

**Published:** 2025-06-06

**Authors:** Franziska Rother, Amishaben R. Parmar, Julia S. Bodenhagen, Letizia Marvaldi, Enno Hartmann, Michael Bader

**Affiliations:** 1 Max-Delbrück-Center for Molecular Medicine (MDC), Robert-Rössle-Str. 10, D-13125 Berlin, Germany; amishabenramanbhai.parmar@unito.it (A.R.P.); ju-bodenhagen@t-online.de (J.S.B.); 2 Institute for Biology, University of Lübeck, Ratzeburger Allee 160, D-23562 Lübeck, Germany; enno.hartmann@uni-luebeck.de; 3 Department of Neuroscience “Rita Levi Montalcini”, Neuroscience Institute Cavalieri Ottolenghi, University of Torino, Via Cherasco 15, 10126 Torino, Italy; letizia.marvaldi@unito.it; 4 German Center for Cardiovascular Research (DZHK), Partner Site Berlin, Hessische Strasse 3-4, D-10115 Berlin, Germany; 5 Charité Universitätsmedizin Berlin, Corporate Member of Freie Universität Berlin and Humboldt-Universität zu Berlin, Charitéplatz 1, D-10117 Berlin, Germany

**Keywords:** importin, karyopherin, motor neuron disease, ADHD, hyperactivity, nucleocytoplasmic transport

## Abstract

Nucleocytoplasmic transport is crucial for neuronal cell physiology and defects are involved in neurodegenerative diseases like amyotrophic lateral sclerosis and Alzheimer’s disease, but also in ageing. Recent studies have suggested, that the classic nuclear import factor adapters KPNA3 (also named importin alpha4) and KPNA4 (also named importin alpha3) could be associated with the development of motor neuron diseases, a condition specifically affecting the neurons projecting from brain to spinal cord or from spinal cord to the muscles. Here we set out to analyze the neuronal function of mice deficient in KPNA3 (*Kpna3*-KO) or KPNA4 (*Kpna4*-KO). The motoric abilities and locomotion at different time points in ageing were tested to study the role of these two genes on motor neuron function. While we did not find deficits related to motor neurons in both mouse models, we discovered a hypermotoric phenotype in KPNA4-deficient mice. Attention deficit/hyperactivity disorder (ADHD) is caused by a combination of genetic, environmental and neurobiological factors and a number of genes have been suggested in genome-wide association studies to contribute to ADHD, including *KPNA4*. Here we provide supportive evidence for KPNA4 as a candidate pathogenic factor in ADHD, by analysing *Kpna4*-KO mice which show ADHD-like symptoms.

## 1. Introduction

Nucleocytoplasmic transport (NCT) defects have been implicated to be key mechanisms in neurodegenerative diseases and a number of recent studies has supported this hypothesis [1]. Most of the current knowledge has been collected from animal models representing diseases like amyotrophic lateral sclerosis (ALS), frontotemporal dementia (FTD), Alzheimer’s disease, or Huntington’s disease, in which scientists have extensively studied NCT components and their impact on the disease development [2,3,4]. Moreover, studies in patients could replicate some of the findings, supporting a crucial function of NCT factors in neuronal health [5,6,7].

The classical nucleocytoplasmic import pathway involves importin β (KPNB1) and importin α (KPNA). In mice, six different KPNA paralogues are existing, while in rats and humans seven paralogues have been found [8,9,10]. Import of a cargo into the nucleus starts with the recognition and binding of its nuclear localization signal (NLS) by KPNA and subsequent binding of KPNA to KPNB1 which then mediates the contact to the nuclear pore. For cargos relying on this classical nuclear import pathway, the presence of KPNA could be crucial to reach the nucleus. KPNA paralogues differ in their cell-specific expression and subcellular localization, and they also display distinct specificities towards their substrates, those proteins which rely on the binding to KPNA in order to enter the nucleus [11,12]. On the other hand, several studies have revealed common cargo proteins between KPNA3 and KPNA4, two paralogues that share a high homology [13,14,15,16]. Besides the classical nuclear import pathway, KPNAs are also involved in intracellular transport processes in neurons. Several studies have demonstrated that KPNA proteins are essential for transport processes along axons [17,18,19,20,21]. Moreover, KPNAs also participate in synapse-to-nucleus signaling in neurons [22,23,24]. These transport events are crucial for neurons to transmit signals (e.g., from a nerve lesion) generated at a distance from the nucleus to the site of transcription in order to adjust to a new situation.

Thus, it is not astonishing that KPNA/KPNB1 mediated transport defects have been implicated in several neurodegenerative diseases. In patients and in mouse models of ALS, which is characterized by a degeneration of both the upper and lower motor neuron, a redistribution of various disease-associated proteins from the nucleus to the cytoplasm has been observed, suggesting a defective NCT [2,25,26,27,28]. Moreover, KPNB1 and KPNA paralogues themselves have been shown to relocate primarily to the cytoplasm in ALS [5,6,7,29]. Overexpression of KPNA4 and KPNA3 could restore the impaired nuclear transport of the protein TDP-43, whose cytoplasmic accumulation in ALS leads to neuronal loss [15]. Additionally, bioinformatics analyses of RNA expression data from spinal cord tissue have shown that KPNA4 and KPNA3 are closely associated with ALS development [25].

Several studies in patients with hereditary spastic paraplegia (HSP), a neurodegenerative disease of the upper motoneuron, have found various missense mutations in the KPNA3 gene, suggesting an underlying defect in nucleocytoplasmic import or a compromised axonal transport [30,31,32]. The authors could show that mutated KPNA3 is downregulated in these patients, has a reduced ability to bind to NLS-bearing cargo proteins and that its translocation into the nucleus is impaired [30]. These studies suggest, that a reduced expression of KPNA3 or its mutation could play a role in the pathogenesis of HSP. Although more than 80 genes have been identified so far being associated with HSP, the pathophysiological mechanisms are all connected to a few biological processes like intracellular transport, formation of cell organelles and mitochondrial function [30]. A key characteristic of motor neurons is that they have very long projections, the axons, which extend from the brain to the spinal cord (upper motor neurons) or from the spinal cord to the muscles (lower motor neurons). The above investigations indicate that KPNA- mediated transport processes are crucial for the proper function of motor neurons and suggest that disruptions in these transport mechanisms may lead to motor neuron dysfunction.

To understand the potential involvement of KPNA3 in the function of motor neurons, the current study aims to investigate the motoric abilities and locomotion in mice deficient in KPNA3. Although much less evidence exists for an impact of the closely related paralogue KPNA4 on the development of motor neuron diseases (MND), we decided to analyse *Kpna4*-knockout (KO) mice in parallel, as KPNA3 and KPNA4 display a high homology and share common cargoes. Using *Kpna4* KO mice, we have shown recently that this KPNA paralogue is involved in pain perception [33]. In order to study the effect of KPNA3 and KPNA4 on motor neuron function and taking into account that MNDs have a quite heterogenous onset—in the case of HSP patients early childhood onsets exist, but also very late onset forms—we investigated young, adult and old mice. A comparable study has not been performed so far and regarding the currently established association of KPNA3 and MND, it should clarify, if a total absence of KPNA3 (or KPNA4) would be sufficient to develop symptoms of motor neuron degeneration.

## 2. Materials and Methods

### 2.1. Ethics Statement

This study was performed according to the current national guidelines for the humane use of laboratory animals and approved by the Landesamt für Gesundheit und Soziales Berlin (No. G0023/22).

### 2.2. Animals

Female and male *Kpna4*-KO mice with wildtype (WT) littermates and female and male *Kpna3*-KO mice with WT littermates were used for this study. 11 mice per group were initially assigned to the tests, a total of 88 mice was used. Calculation of the sample size was based on the performance in the rotarod test as a primary outcome. For the calculation of the sample size Student’s *t*-test (α = 0.05, power 80%) was used, as no information was available for estimated correlations regarding the age. The calculation was performed on the basis of earlier publications of changes in rotarod test in mice with motor neuron diseases [34,35,36]. Exclusion criteria were not set explicitely, but mice not fulfilling the health criteria during a daily control were not used in experiments. The decline of group sizes during ageing occurred due to sickness or death of mice for unknown reasons. The exact group numbers are stated in each experiment. The age of the mice was 3 months, 8 months, 12 months and 18 months. Generation of these mice has been described earlier [37,38]. Mice were kept at 22.0 ± 0.2 °C in a humidity-controlled room under a 12-h light–dark cycle with free access to food and water. During the experiments, experimentators were not blinded regarding the genotype of mice, as the obtained outcomes were objective measurements done by the behaviour setup (e.g., open field). For the analysis of hindlimb clasping videos, a blinding was used. A protocol was prepared before the study started, this was part of the animal application. It was registered at https://www.animaltestinfo.de (accessed on 4 June 2025).

### 2.3. Assessment of Motoric Abilities and Locomotion

Tests were performed on 2 consecutive days. Day one started with the open field test. One hour after the open field test had been finished, mice were placed on the Rotarod for training. The next day, mice were subjected to the inverted screen test. One hour after this test had been finished, mice were placed on the Rotarod for the Rotarod test. The tail suspension test was performed on a different day.

Tail suspension reflex test. The test serves as a marker of disease progression in a number of mouse models of neurodegeneration. Mice were suspended by the tail for 15 s at 20 cm above an examination area and the posture of the hindlimbs was examined. Clasping was defined as a retraction of hindlimbs, so that they touch the abdomen for more than 3 s. To assess a possible disease progression more precisely, videos were taken from 8, 12 and 18 months old mice and the posture of hindlimbs was assessed by a scoring system [39]. Briefly, if hindlimbs were consistently stretched away from the abdomen, this was considered score 0; if one hindlimb was partially retracted towards the abdomen for more than 50% of the time, this was considered score 1; if both hindlimbs were partially retracted towards the abdomen for more than 50% of the time suspended, this was considered score 2; if the hindlimbs were completely retracted towards the abdomen for more than 50% of the time, this was considered score 3. Three videos per mouse were recorded and analysed.

Rotarod test. Mice were placed on an accelerating rotarod to investigate motor coordination. The rotarod started at 4 rpm speed and accelerated progressively up to 40 rpm during 5 min. One day before the test, mice received a training consisting of a habituation to the rotarod with 4 rpm for 5 min and two sessions of accelerating rotarod for 5 min. On the test day, the latency to fall was recorded in three consecutive trials.

Kondziela’s inverted screen test. Mice were placed in the middle of a 43 cm square wire mesh grid consisting of 12 mm wide squares with 1 mm wire diameter. The grid was surrounded by a plexiglas boundary preventing the mice from climbing on the other side of the grid. After placing the mice on the screen, the screen was turned top-to-bottom within 2 s so that the mice had to hold their own weight. The distance to the padded surface was 50 cm. The latency to fall was recorded within 120 s, after 120 s the test was stopped.

Open field test. We used the ActiMot system and Actimot software (TSE systems, version 8.10.0.0) for assessing mouse locomotion. Mice were placed in 480 × 480 mm transparent arenas and the locomotion was recorded for 60 min in the open field after a period of 10 min of habituation. Activity (defined by a locomotion velocity >5 cm/s), hyperactivity (defined by a locomotion velocity >20 cm/s) and resting time were assessed, as well as distance travelled, locomotion velocity (calculated as distance in activity time divided by activity time) and time spent in the center.

### 2.4. Statistical Analysis

The data were calculated and displayed as mean ± SEM using GraphPad Prism7 software. Statistical analysis was performed by Two-way ANOVA with Sidak’s test for correction of multiple comparisons. For the analysis of hindlimb clasping in 3 months old mice, Fisher’s exact test was used. Significance levels were * *p* < 0.05, ** *p* < 0.01, *** *p* < 0.001, **** *p* < 0.0001.

## 3. Results

**Figure 1:**
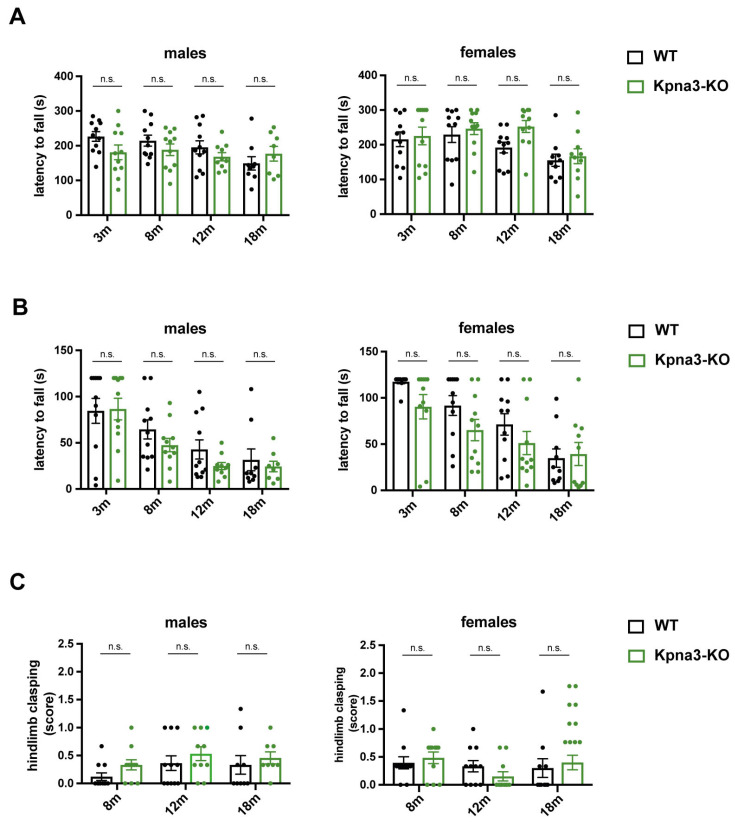
Analysis of neuromuscular function in KPNA3-deficient mice throughout ageing. Female and male *Kpna3*-KO mice and C57Bl/6 wild-type (WT) mice of different ages (3 months (m): n = 11; 8 m: n = 11; 12 m: n = 10–11; 18 m: n = 8–10) were submitted to rotarod test (**A**), inverted screen test (**B**) and tail suspension test (**C**). n.s.: not significant.

We performed a panel of different tests in KPNA3-deficient mice over their lifespan in both sexes. Rotarod tests showed a normal latency to fall compared with age-matched wildtype mice (Figure 1A). The inverted screen test revealed an age-dependent trend of decline in the latency to fall in female and male *Kpna3*-KO and WT mice (Figure 1B). However, no differences were observed when *Kpna3*-KO and WT mice were compared. Likewise, the tail suspension test, assessing the clasping of hindlimbs as a sign of motoric dysfunction, showed no pathological clasping in *Kpna3*-KO mice compared with WT (Table 1, Figure 1C).

**Figure 2:**
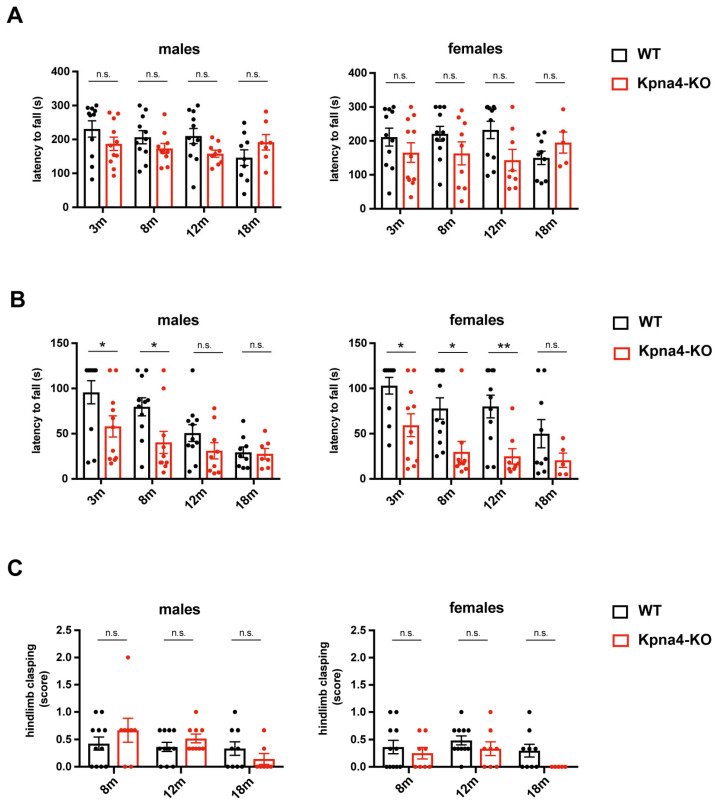
Analysis of neuromuscular function in KPNA4-deficient mice throughout ageing. Female and male *Kpna4*-KO mice and C57Bl/6 WT mice of different ages (3 months (m): n = 11; 8 m: n = 9–11; 12 m: n = 8–11; 18 m: n = 5–9) were submitted to rotarod test (**A**), inverted screen test (**B**) and tail suspension test (**C**). n.s.: not significant. Significance levels were * *p* < 0.05, ** *p* < 0.01.

As KPNA4 dysfunction has in various studies been implicated in the pathophysiology of ALS, a neurodegenerative disease affecting the upper and the lower motoneuron, we assessed the neuromuscular function in KPNA4-deficient mice over their lifespan in both sexes. The rotarod test showed no difference in the latency to fall in male and female *Kpna4*-KO mice when compared to age-matched WT littermates (Figure 2A). Interestingly, in the inverted screen test, KPNA4-deficient mice had a clear reduction in the latency to fall which was apparent in males and females, while the tail suspension test did not reveal any abnormal hindlimb clasping (Figure 2B,C, Table 1). The discrepancy of the abnormal inverted screen test and the normal rotarod test let us assume that instead of a neuromuscular deficiency, the motoric behavior could be changed in *Kpna4*-KO mice.

**Figure 3:**
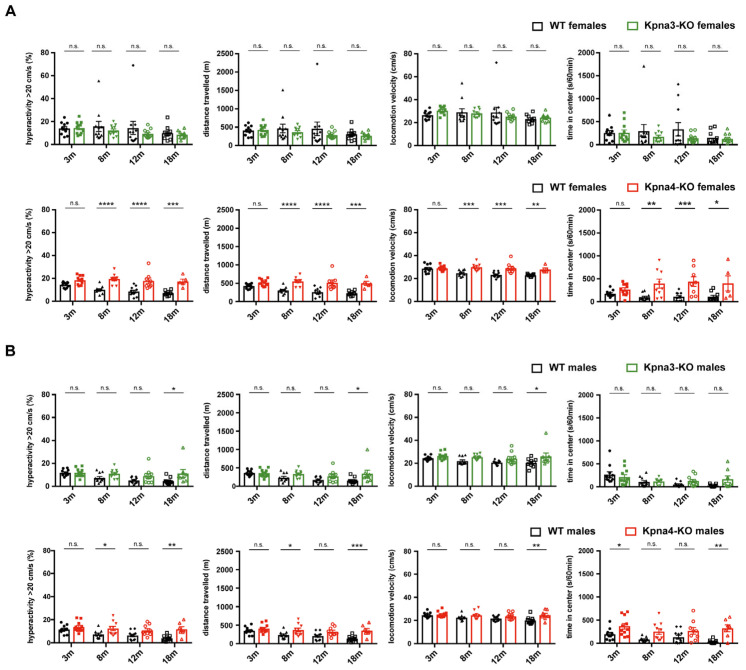
Spontaneous locomotion in KPNA3- and KPNA4-deficient mice. Female and male *Kpna3*-KO mice, *Kpna4*-KO mice and C57Bl/6 WT mice of different ages (3 months (m): n = 11; 8 m: n = 9–11; 12 m: n = 8–11; 18 m: n = 5–11) were submitted to open field experiments for 60 min with a habituation phase of 10 min. Female *Kpna4*-KO mice but not *Kpna3*-KO mice clearly show hyperactivity (defined as part of the time when locomotion velocity is >20 cm/s) and increased distance travelled starting at 8 months of age, as well as increased locomotion velocity (**A**). In male *Kpna4*-KO mice, enhanced hyperactivity and distance travelled could be found at 8 and 18 months of age, while at 12 months no significant effect was shown (*p* = 0.09 for hyperactivity; *p* = 0.07 for distance travelled) with no clear effect on locomotion velocity. The time in the center was significantly increased for several time points in female and male *Kpna4*-KO mice, but not for *Kpna3*-KO mice (**A**,**B**). n.s.: not significant. Significance levels were * *p* < 0.05, ** *p* < 0.01, *** *p* < 0.001, **** *p* < 0.0001.

Next, we measured spontaneous locomotion in our KPNA3- and KPNA4-deficient mouse lines using an open field arena. Here, we found increased activity levels in *Kpna4*-KO mice, leading to an increased distance travelled and a higher locomotion velocity. While the effects were mild in *Kpna4*-KO males, *Kpna4*-KO females displayed striking differences in spontaneous locomotion compared to WT, becoming apparent at 8 months of age which persisted also in old mice (Figure 3A,B). Besides this hypermotoric behaviour, female and male *Kpna4*-KO mice spent more time in the center of the open field arena, revealing an increased exploratory behaviour compared to WT. In contrast to *Kpna4*-KO mice, neither male nor female *Kpna3*-KO mice showed signs of enhanced activity or exploratory behaviour (Figure 3A,B), albeit including one very active animal in the male *Kpna3*-KO group resulted in a statistical significance (*; *p* = 0.0182; 0.0158 and 0.0264 respectively). Thus, we conclude, that *Kpna4*-KO mice, but not *Kpna3*-KO mice develop a hypermotoric phenotype in adulthood, while motoneuron function is not affected.

## 4. Discussion

Neurodegenerative diseases are defined by a progressive loss of neurons and many different forms are existing. Despite the heterogeneity of these diseases and their symptoms, in part attributed to the subtype and localization of degenerating neurons, studies have established some common mechanisms of neurodegeneration, one of them being a defective NCT [2]. Extensive research on ALS, as well as on FTD has suggested numerous factors as causal candidates involved in nucleocytoplasmic shuttling for both diseases, including KPNB1 [29,40,41,42,43]. Other studies have shown a clear role for KPNA/KPNB1-mediated nuclear transport in disorders like HSP or spinocerebellar ataxia, a disease characterized by atrophy of the cerebellum and progressive loss of coordination [30,44]. However, the recent progress in research has not yet been able to clarify, if and how exactly the malfunction of NCT can serve as the underlying cause for neurodegenerative diseases or in which way the cellular degenerative processes themselves (e.g., mislocalization of abnormal proteins, formation of membrane-less granules) contribute to a decline in the overall capacity of NCT or to the specific disruption of single transport factors. Moreover, it is still a matter of debate, which of the multiple and complex events of NCT are dysregulated in neurodegenerative diseases like ALS or HSP, as different factors are involved in import from cytoplasm into the nucleus and export from nucleus to the cytoplasm. As an example, while it could be shown that overexpression of KPNB1 reversed the cytoplasmic sequestration of disease-associated proteins in ALS, there have been controversial results for the role of KPNA paralogues [15,45,46].

In the current study we aimed to clarify, if the presence of KPNA3 is indispensable for the normal function of motor neurons. The motoric behaviour and spontaneous locomotion of KPNA3-deficient mice were assessed at different time points throughout life using a panel of tests. Interestingly, non of these tests revealed abnormalities in motor neuron function and locomotion behaviour in mice deficient for KPNA3. Thus, we cannot confirm the earlier established link between mutation or reduced expression of KPNA3 and MND. We conclude that nuclear import involving KPNA3/KPNB1 seems to be dispensable for a proper motor neuron function and locomotion behaviour in young, adult and aged mice.

Recent results of studies in ALS mouse models have suggested that nuclear export rather than import processes could play a role in disease pathophysiology [40]. Transgenic mice with a mutation in the SOD1 gene (SOD1G93A), the same mutation which can be found in ALS patients, relocate the mutated SOD1 form to the cytoplasm. Hence, it could be shown, that wildtype SOD1 is still normally distributed between nucleus and cytoplasm, raising the hypothesis, that nuclear exclusion of mutated SOD1 could be a protective mechanism to prevent accumulation of misfolded SOD1 in the nucleus [47].

The investigation of *Kpna4*-KO mice in the same panel of tests revealed interesting results: while the motoric function was not affected in the rotarod test and in the tail suspension test, we found a clear reduction in the latency to fall in the inverted screen test in young and adult female, and in young and adult male mice. The inverted screen test, published by Kondziela in 1964, measures the muscle strength using all four limbs. However, in this test it is difficult to distinguish between a true muscle weakness and other reasons to „fall“ from the grid. A lower latency to fall could also be attributed to a lower attention level of mice which would lead to less carefully gripping the wire mesh. Another option could be a lower level of fear resulting in „jumping“ onto the padded surface or a decreased motivation to hold the own weight. Interestingly, we did not observe a statistical significance in this test in old mice (albeit a tendency was found in females, supporting our findings). The reason for this could be a natural decline in the ability to hold the own body weight in aged mice, which we observed in WT females and males.

Analysis of *Kpna4*-KO mice in the open field test revealed indeed a difference in locomotion behaviour in female mice: adult females showed a striking hyperactivity in the open field, that was consistently found in adult and aged (8, 12 and 18 months) females. This hyperactivity was partially also detected in male 8 and 18 months old *Kpna4*-KO mice albeit to a lesser extent. Not consistently with this finding, the male mice aged 3 and 12 months did not show differences in activity compared to WT. While the locomotion behaviour in young mice was similarly normal in female and male mice, we currently cannot explain the finding in 12 months old male mice. Our data show that the hyperactivity effects are in general much more pronounced in female *Kpna4*-KO mice. Analysis of the distance travelled and locomotion velocity supported the detected increased locomotion raising the hypothesis of an attention deficit/hyperactivity disorder (ADHD)– like phenotype in *Kpna4*-KO mice. An ADHD-like phenotype would indeed hamper the inverted screen test as has been shown recently in a mouse model of ADHD, where the mice did not show significant differences in the wire hanging, rotarod and grip strength tests, but a reduced latency to fall in the inverted screen [48]. Thus, we conclude, that absence of KPNA4 could be related to the development of an ADHD-like phenotype. In an earlier study we have shown an increased homecage activity in *Kpna4*-KO mice during the dark period but a normal activity in the open field test, however, this study had used 2–5 months old male mice [23]. We could now reproduce the normal locomotion in young *Kpna4*-KO male mice but also showed that both sexes at adult age present significantly increased locomotor activities in the open field as shown by enhanced hyperactivity and an increased distance travelled. Importantly, the effect was much stronger in females than in males but persisted in both sexes during ageing. It has been shown earlier that in locomotion experiments female mice travel significantly longer distances compared to male mice (up to 30%) [49]. On the other hand, a recent study comparing the motoric behaviour of male and female mice did not find significant differences in the open field test [50]. Interestingly, both investigations were performed on C57/Bl6 mice using an open field system. Thus it is still a matter of debate if the sex contributes to motoric behaviour or not and if so, in which conditions. In our study we could not observe a statistically significant sex effect in the distance travelled in the open field in WT mice. Thus, the sex of mice seems not to have an influence on the activity in the open field in the control groups and the sex differences found in *Kpna4*-KO mice are related to the *Kpna4* genotype. Interestingly, *Kpna4*-KO mice also spent more time in the center of the open field arena compared to WT littermates, and this effect again was more pronounced in female mice. These results stand in contrast to a recent study showing normal locomotion and increased anxiety-related behaviour in *Kpna4*-KO mice [51]. However, the study of Sakurai et al. only tested very young (8–10 weeks) and only male animals.

ADHD is a neurodevelopmental disorder characterized by overactivity, impulsivity and a lack of attentivity starting at childhood and often persisting until adulthood [52]. It affects 2–7% of children with a preference for males which disappears in adult age. Recent changes in diagnostic criteria coupled with a higher awareness of ADHD have led to an increased prevalence throughout the last years. There is a strong (poly)genetic component in the pathogenesis of the disorder, but the responsible genes are still elusive. In a recent genome-wide association study metaanalysis (GWAS-MA) including 17,149 ADHD cases and 32,411 controls 4 genome-wide significant variants were found to be linked to ADHD over the whole lifespan [53]. One of these SNPs was rs1920644 located on chromosome 3 close to the KPNA4 gene and associated with the expression of this gene [53].

Our data together with the GWAS-MA strongly support *KPNA4* as a candidate gene for ADHD which affects both sexes. The pronounced phenotype observed in female *Kpna4*-KO mice compared to males seem to stand in contrast with the current knowledge about ADHD in humans, which is more often diagnosed in boys compared to girls (ratio 2.4:1) [52]. However, it is also known that ADHD is largely underdiagnosed in girls due to different presentation of the disease. While boys often present with a hyperkinetic phenotype and impulsiveness, attracting the attention of parents and teachers, girls often present with an inattentive phenotype which is less disruptive for the environment and therefore leads to lower rates (and later timepoints) in diagnosis and treatment [54]. Finally, with human ADHD being a polygenetic condition, requiring the interaction of multiple genes, *KPNA4* is only one factor contributing to the phenotypic presentation of this disease.

We believe that the ADHD-like phenotype found in *Kpna4*-KO mice is subclinical in young mice and becomes obvious when they start to reach adulthood. In that light, one could only speculate that the observed hyperactive phenotype is related to neuronal transformations occuring during ageing as a direct effect of KPNA4 absence. Further analysis in adult and aged mice are necessary and will clarify the role of KPNA4 in the development of hyperactive behaviour.

## Figures and Tables

**Table 1:** Hindlimb clasping in three months old KPNA3- and KPNA4-deficient mice. 3 months old mice were analyzed for hindlimb clasping in the tail-suspension-test. *p*-values were determined using Fisher’s exact test.

	Hindlimb Clasping (per Number of Mice in Group)	*p*-Value
*Kpna3*-WT females	0/11	>0.9999
*Kpna3*-KO females	1/11	
*Kpna3*-WT males	0/11	>0.9999
*Kpna3*-KO males	0/11	
*Kpna4*-WT females	0/11	0.4762
*Kpna4*-KO females	1/10	
*Kpna4*-WT males	0/11	>0.9999
*Kpna4*-KO males	1/11	

## Data Availability

All obtained data are part of the manuscript.

## Abbreviations

The following abbreviations are used in this manuscript:

ADHD	Attention deficit/hyperactivity disorder
NCT	Nucleocytoplasmic transport
ALS	Amyotrophic lateral sclerosis
FTD	Frontotemporal dementia (FTD)
HSP	Hereditary spastic paraplegia
MND	Motor neuron disease
WT	Wildtype
GWAS-MA	Genome-wide association study metaanalysis
